# Evidence for the Link Between KK-42 and the *DH-PBAN* Gene in Two Silkmoth Species, with Impacts on Developmental Traits

**DOI:** 10.3390/biology15070542

**Published:** 2026-03-28

**Authors:** Haixu Bian, Yufeng Lin, Yuping Li, Jingchen Sun, Yanqun Liu

**Affiliations:** 1 College of Animal Science, South China Agricultural University, Guangzhou 510642, China; bianhaixu666@163.com (H.B.);; 2 College of Agriculture and Horticulture, Liaoning Agricultural Vocational and Technical College, Yingkou 115009, China; 3 Department of Sericulture, College of Bioscience and Biotechnology, Shenyang Agricultural University, 120 Dongling Road, Shenyang 110866, China

**Keywords:** silkmoth, KK-42, *DH-PBAN* gene, sequence comparison, expression patter

## Abstract

Diapause hormone (DH) is an important endocrine substance capable of influencing diapause in Lepidoptera moths that is encoded by the neuropeptide hormone *DH-PBAN* gene. Imidazole derivative KK-42 is a synthetic insect growth regulator that can affect diapause in Lepidoptera moths, and appears to have an opposite physiological function to DH. To test the hypothesis that KK-42 may be operating through DH to affect diapause, here, we used two Lepidoptera species *Bombyx mori* L. and *Antheraea pernyi* that enter egg and pupal diapause, respectively, through examining whether KK-42 can influence *DH-PBAN* and some associated mRNA expression. We found that the protein sequences of *DH-PBAN* in insects were highly variable, although the PRXamide C-terminus was conserved. We also found that KK-42 induced significant up-regulation and prolonged expression duration of *DH-PBAN* in both *A. pernyi* and *B. mori* pupae, as well as in trimolter larvae of *B. mori* that were induced by the application of KK-42 from the normal tetramolter larvae. In addition, KK-42 can significantly upregulate glutamic acid decarboxylase (*GAD*) expression in *B. mori* in transcriptome data. Our findings suggested that KK-42 influences diapause by upregulating *GAD* expression, promoting DH accumulation to prolong the secretion time of *DH-PBAN*.

## Simple Summary

Diapause hormone (DH), an essential endocrine factor regulating insect diapause, is encoded by the neuropeptide hormone *DH-PBAN* (diapause hormone-pheromone biosynthesis activating neuropeptide) gene. The imidazole derivative KK-42, as an insect growth regulator, has been demonstrated to influence diapause in Lepidoptera moths. This research reveals that the protein sequence of DH-PBAN, which is encoded by the corresponding gene in insects, exhibits significant variability. Specifically, KK-42 modulates diapause by upregulating the expression of the *GAD* gene, thereby promoting the accumulation of DH and extending the secretion duration of the *DH-PBAN* gene. These findings provide experimental evidence for the functional association between KK-42 and the *DH-PBAN* gene.

## 1. Introduction

Diapause hormone (DH) is a member of the FXPRLamide neuropeptide (NP) family first identified in the domestic silkmoth *Bombyx mori* L. (Lepidoptera: Bombycidae) [1]. DH is synthesized in seven pairs of neurosecretory cells of the mother’s subesophageal ganglion (SG) [2,3] and is directly responsible for the induction of egg diapause of *B. mori* [1]. Injection of DH can induce *B. mori* multivoltine strain Nistari that produces non-diapause eggs in natural conditions to produce diapause eggs [4]. DH in *B. mori* is encoded by the *DH-PBAN* gene that also encodes pheromone biosynthesis activating neuropeptide (PBAN) and three short neuropeptides, α-, β- and γ-SGNP [5]. *DH-PBAN* homologous genes are expressed in many insect species other than *B. mori*; however, DH only exhibits diapause induction effect in *B. mori* [6]. In contrast, in the *Helicoverpa/Heliothis* complex and the bamboo borer, *Omphisa fuscidentalis* Hampson (Lepidoptera: Crambidae), DH effectively breaks pupal diapause and larval diapause, respectively [7,8]. The expression analysis indicated that at the early stage of pupal-adult development of B. mori, the *DH-PBAN* mRNA was 2.7 times higher in the diapause type SG than in the non-diapause SG [9]. However, in the Chinese oak silkmoth *Antheraea pernyi* (Guérin-Méneville) (Lepidoptera: Saturiidae) that enters pupal diapause, the *DH-PBAN* mRNA is much more abundant in non-diapausing pupae than diapausing counterparts [10]. Like *A. pernyi*, the *Helicoverpa/Heliothis* complex that enters pupal diapause is also present in this case [11].

KK-42 (1-benzyl-5-[(E)-2,6-dimethyl-1,5-heptadienyl] imidazole) is an imidazole derivative that functions as a growth regulator of insects [12]. KK-42 can induce precocious metamorphosis when applied to larvae of *B. mori* [13], *A. pernyi*, the fresh fly *Sarcophaga bullata* Parker (Diptera: Sarcophagidae) [14], the European corn borer *Ostrinia nubilalis* Hübner (Lepidoptera: Crambidae) [15], and affect the development of the grasshopper *Locusta migratoria* L. (Orthoptera: Acrididae) [16], the locust *Schistocerca gregaria* Forskal (Orthoptera: Acrididae) [17], and *Tenebrio molito* L. (Coleoptera: Tenebrionidae) [18]. KK-42 has been shown to completely terminate the egg diapause (pharate first-instar larvae) in wild silkmoth *Antheraea yamamai* (Guérin-Méneville) [19,20] and the gypsy moth *Lymantria dispar* L. (Lepidoptera: Erebidae) [21,22]. KK-42 also has the ability to reduce the incidence of egg diapause in progeny of *B. mori* that are programmed to produce diapausing eggs [23]. Our previous study further showed evidence that KK-42 can retard the termination of pupal diapause when applied to diapausing pupae in *A. pernyi* and the corn earworm *Helicoverpa zea* Boddie (Lepidoptera: Noctuidae), and boost pupal diapause incidence when administered to the final instar larvae of *H. zea* and the fresh fly *Sarcophaga crassipalpis* Macquart (Diptera: Sarcophagidae) [24]. Taken together, KK-42 seems to have an opposite physiological function to DH.

Previous studies support the hypothesis that KK-42 may terminate diapause by acting through DH [25]. To comprehensively investigate this link and explore whether KK-42′s effect on *DH-PBAN* is conserved across different diapause programs, we employed a comparative strategy using two silkmoth models. We utilized the domestic silkworm, *B. mori*, which undergoes embryonic diapause and possesses unparalleled genomic and genetic resources, enabling detailed mechanistic inquiry. In parallel, we examined the Chinese oak silkmoth, *A. pernyi*, a well-established model for pupal diapause. This comparative approach allows us to distinguish conserved, core effects of KK-42 on *DH-PBAN* expression from those specific to a particular diapause stage or species. Our experiments demonstrate that KK-42 consistently upregulates *DH-PBAN* mRNA in both species, thereby establishing, for the first time, a direct chemical regulatory link between KK-42 and this critical neuropeptide gene across divergent diapause contexts. Our findings provide a theoretical basis for developing KK-42 into a molecular tool for regulating insect development and diapause to boost sericulture efficiency, while also elucidating the conserved regulatory mechanism of the insect growth regulator KK-42 on the diapause regulatory gene *DH-PBAN* in Lepidoptera with distinct diapause strategies, and addressing the critical gap in the functional association between KK-42 and the DH-PBAN neuroendocrine pathway.

## 2. Materials and Methods

### 2.1. Insects

The polyvoltine strain Nistari (non-diapause) and bivoltine strain Qiubai of *B. mori* (laboratory-reared for 4 consecutive generations, F4), and monovoltine strain Yuda no. 1 of *A. pernyi* (from the 2nd filial generation, F2) were used in this study. The eggs of *B. mori* were kindly provided by the Silkworm Genk Bank of Southwest University, China and the pupae of *A. pernyi* were from the Sericulture Institute of Henan Province, China, respectively. The larvae of *B. mori* were reared indoors with fresh mulberry leaves at 25 ± 1 °C under the daily light (LD 12:12), and the resulting pupae were also stored in the same conditions. The diapausing pupae of *A. pernyi,* as a semi-domesticated insect reared on the *Quercus liaotungensis,* were stored at 25 ± 1 °C under a dark environment to maintain the diapause status. To promote diapause termination of *A. pernyi*, the diapausing pupae were transferred to a light incubator (RDN-260B-4, Ningbo Ledian Instrument Manufacture Co., Ltd., Ningbo, China) at 25 ± 1 °C under an LD 17:7 photoperiod.

### 2.2. Chemicals and Application

The imidazole-based insect growth regulator KK-42 (1-benzyl-5-[(E)-2,6-dimethyl-1,5-heptadienyl] imidazole) was synthesized as previously described [13], and the activity to induce precocious metamorphosis in *B. mori* larvae was confirmed [24]. Before each experiment, the stock solution was diluted with 50% acetone aqueous solution to the required working concentrations: 1 μg/μL for topical application to *B. mori* larvae, 2 μg/μL for microinjection into *B. mori* pupae, and 10 μg/μL for microinjection into *A. pernyi* pupae. All solutions were freshly prepared on the day of the experiment to ensure biological activity. The newly-ecdysed 4th instar larvae of *B. mori* received a 1 μL topical application to the dorsal surface of KK-42 solution (final concentration: 1 μg). The KK-42 solution was injected into developing pupae of *B. mori* at day 1 (final concentration: 2 μg) and diapausing pupae of *A. pernyi* (final concentration: 100 μg), respectively. Acetone served as the control. Prior to the injection of KK-42, 20 µg of 20-Hydroxyecdysone (20E) (D&B Biological Science and Technology Co., Ltd., Shanghai, China) dissolved in 10% ethanol was injected into diapausing pupae to accelerate the diapause termination process of *A. pernyi* pupae. The concentrations of KK-42 used in this study were selected based on previously published effective doses for the corresponding species and developmental stages [13,24].

### 2.3. SG Collection

Suboesophageal ganglia (SG) were carefully sampled in the precooled insect Ringer physiological saline buffer under a dissecting microscope (20×; Olympus Optical, JM, 43-2 Hatagaya 2-chome, Shibuya-ku, Tokyo, Japan). For *A. pernyi*, we collected SGs from 60 diapausing pupae at 24 h, 48 h, 96 h, 144 h and 192 h after KK-42 treatment. For *B. mori*, 4th instar larvae (*n* = 60) and 180 pupae (90 for treatment group, 90 for control group, including 45 Nistari and 45 Qiubai), we collected SGs every 24 h after KK-42 treatment until the next developmental stage. For each sampling time point, 3 biological replicates were set, with each replicate consisting of 3–5 individual insects. The obtained SGs were immediately placed into 200 μL TRIzol reagent (Beijing Aidlab biotechnologies Co., Ltd., Beijing, China) and stored at −80 °C prior to RNA extraction.

### 2.4. RNA Isolation

Total RNA was isolated from a single SG with TRIzol reagent and dissolved in 10 μL ddH_2_O. RNA integrity and concentration were checked with a NanoDrop ND-1000 spectrophotometer (Thermo Scientific, Wilmington, DE, USA). One microgram of total RNA was used to synthesize the cDNA using a PrimeScriptTM RT Reagent Kit with gDNA Eraser (Perfect Real Time) (TaKaRa Biotechnology Dalian Co., Ltd., Dalian, China).

### 2.5. RNA-Seq

At 72 h post-injection, all silkworm pupae from each group were dissected, and SG samples were collected. Resulting in a total of six samples: three KK-42 treated samples (KKSG1, KKSG2, KKSG3) and three control samples (CKSG1, CKSG2, CKSG3). Total RNA (~1 μg) was used as input material for individual RNA sample preparation. Sequencing libraries were generated using NEBNext UltraTM RNA Library Prep Kit for Illumina (NEB, San Diego, CA, USA) following the manufacturer’s recommendations and index codes were added to attribute sequences to each sample. First-strand cDNA was synthesized using random hexamer primer and second-strand cDNA synthesis was subsequently performed using DNA Polymerase I and RNase H. Library quality was assessed on the Agilent Bioanalyzer 2100 system. Finally, pair-end sequencing was performed with a read length of 2 × 150 bp on the Illumina Hiseq 2500 sequencing platform.

High-quality clean reads were obtained after removing the adaptor contamination, low-quality bases, and undetermined bases. Trimmomatic was used to process the raw data to remove low-quality reads [26]. The remaining clean reads were mapped to the latest version of the silkworm genome (genome sequences and annotation file downloaded from SilkDB 3.0, https://silkdb.bioinfotoolkits.net/doc/download.html, accessed on 10 January 2026) using HISAT2 (v2.4) [27]. Only reads with a perfect match or one mismatch were further analyzed and annotated based on the reference genome. Hisat2 tools were used to map with the reference genome [28]. To ensure the quality of the transcriptome library, we used RSeQC to test gene coverage, sequencing saturation and sequence distribution, respectively [29]. Differential gene expression analysis across groups (CKSG vs. KKSG) was performed using DESeq2_1.50.2 software [30]. Genes with a false discovery rate (FDR) ≤ 0.05 and a fold change (FC) of |log2FC| > 1 were considered differentially expressed genes (DEGs). Additionally, all DEGs were subjected to Gene Ontology (GO) and Kyoto Encyclopedia of Genes and Genomes (KEGG) analyses [31,32].

### 2.6. Quantitative Real-Time PCR (qRT-PCR) Analysis

The gene-specific primer pairs for qRT-PCR are shown in Table 1. The *rp49* gene was used as the internal control for both *A. pernyi* and *B. mori* samples. Primers were designed with Integrated DNA Technologies (Coralville, IA, USA) online server (https://www.idtdna.com). The qRT-PCR analysis was performed on Bio-Rad CFX Connect Real-Time System (Hercules, CA, USA) with a 10 μL reaction volume containing 3.6 μL of TB GreenTM Premix Ex TaqTM (Tli RNaseH Plus, TaKaRa, Kusatsu, Japan), 0.4 μL of specific primers (10 μM), 1 μL of cDNA and 5 μL of ddH_2_O. The qRT-PCR program was initiated at 95 °C for 40 s, followed by 40 cycles at 95 °C for 5 s and 60 °C for 1 min, and a final stage of 60–95 °C to determine melting curves of the amplified products. Each reaction was repeated with four independent biological replicates. The gene expression level was depicted using 2^−ΔΔCq^ values [33]. The statistical analysis was tested by Student’s *t*-test in SPSS 18.0 software, using a *p*-value of *p* < 0.05 as the threshold for significance.

### 2.7. Sequence Comparison and Phylogenetic Analysis

To obtain the *DH-PBAN* homolog, we used the amino acid sequence of the DH-PBAN precursor protein encoded by the *DH-PBAN* gene from *A. pernyi* to search against NCBI database. We also searched by gene name (pban for non-Diptera species) in the website InsectBase 2.0 [36] to get the PBAN-type neuropeptide sequences. CAPA used as an outgroup reference sequence is a highly conserved neuropeptide family in insects, belonging to the FXPRLamide superfamily, which encodes products that exert physiological functions by mediating the ERK signaling cascade, and it is a widely used outgroup reference sequence for phylogenetic analysis of insect neuropeptide systems [37]. The *DH-PBAN* homologous sequences were aligned by MUSCLE with MEGA X v10.2.1 [38] and edited by hand. The phylogenetic relationship was built based on the amino acid sequence in MEGA X v10.2.1 with the neighbor-joining method by 1000 bootstrap replications.

## 3. Results

### 3.1. Structure Change of DH-PBAN Genes in Insects

**Figure 1:**
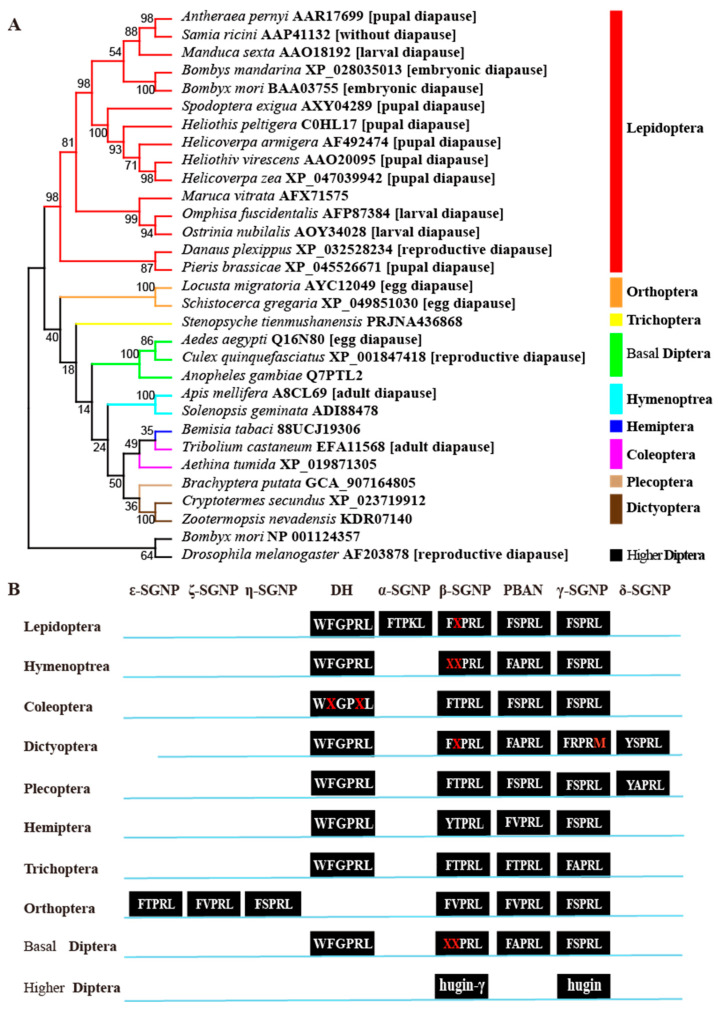
Phylogenetic relationship (**A**) and conserved FXPRLamide structure (**B**) of *DH-PBAN* peptides. (**A**) Maximum-likelihood phylogenetic tree of insect DH-PBAN-related peptides. Branches with bootstrap support below 50% were collapsed to form polytomies, reflecting uncertainty in deep nodal relationships. Support values above 50% are shown at the nodes. The diapause type (embryonic, larval, or pupal) for each species is indicated in brackets. Accession numbers follow species names. Sequences from *B. mori* and *Drosophila melanogaster* CAPA peptides were used as outgroups. (**B**) Sequence logo of the conserved C-terminal FXPRLamide motif across the aligned DH-PBAN family peptides. The red font in the legend indicates the conserved functional FXPRLamide C-terminal motif. The full multiple sequence alignment, including all variable regions and enzyme cleavage sites, is provided in Supplementary Figure S1.

Here, we examined the structure of the *DH-PBAN* gene in non-Diptera insects. In the website InsectBase 2.0, we got 392 PBAN-type neuropeptide sequences from eight insect orders, including Lepidoptera, Hymenoptera, Diptera, Coleoptera, Dictyoptera, Plecoptera, Hemiptera and Trichoptera. When we used the *DH-PBAN* pre-prohormone of *A. pernyi* (AAR17699) to search against NCBI database with BlastP, we also found the presence of the *DH-PBAN* homolog in Orthoptera. Some representative *DH-PBAN* sequences were used to characterize the structure of the *DH-PBAN* gene (Figure S1). In the phylogenetic tree (Figure 1A), the relationship of these *DH-PBAN* sequences agreed well with the taxonomical classification, and the *DH-PBAN* sequences from an order formed a separate cluster. Multiple sequence alignment indicated that the protein sequences of the DH-PBAN precursor protein in non-Diptera insects have also undergone significant change among insect orders, as found in Diptera [37].

In these examined insect species across eight orders, nine FXPRLamide neuropeptides were found, including DH, PBAN, α-SGNP, β-SGNP, γ-SGNP, δ-SGNP, ε-SGNP, ζ-SGNP and η-SGNP, all of which were followed by the endoproteolytic cleavage sites (G-R, G-K-R, G-R-R). Four novel NPs were temporarily named as δ-SGNP, ε-SGNP, ζ-SGNP and η-SGNP, respectively (Figure 1B). In Lepidoptera, all *DH-PBAN* genes share similar structural characteristics and encode proteins that are cleaved into five FXPRLamide family neuropeptides: DH, PBAN, α-SGNP, β-SGNP and γ-SGNP. Among these nine insect orders, PBAN, β-SGNP and γ-SGNP are highly conserved in the FXPRLamide C-terminus, while α-SGNP is Lepidoptera-specific. In the grasshopper *L. migratoria* (Orthoptera), six FXPRLamide C-terminus (X = T, V or S) sequences were found, but without the coding sequences for DH [39,40]. These results showed that the protein sequences of *DH-PBAN* gene in insects were highly variable, although the PRXamide C-terminus was conserved.

### 3.2. Effect of KK-42 on DH-PBAN Gene in A. pernyi Diapause Pupae

**Figure 2:**
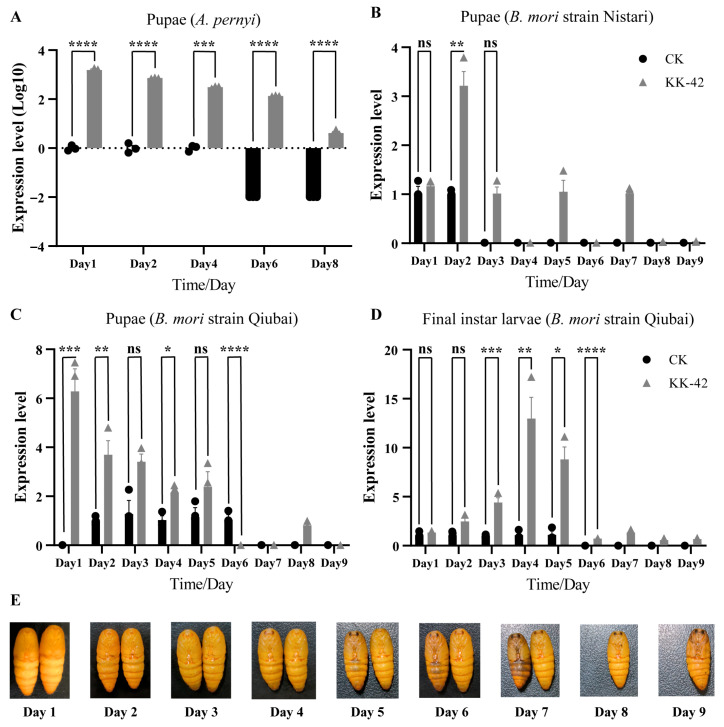
Expression changes of *DH-PBAN* mRNA in SG after KK-42 treatment (biological replicates, *n* = 3). (**A**) Relative expression levels in pupae of *A. pernyi* monovoltine strain Yuda No. 1. (**B**) Relative expression levels in pupae of *B. mori* non-diapause strain Nistari. (**C**) Relative expression levels in pupae of *B. mori* diapause strain Qiubai. (**D**) Relative expression levels in the final instar trimolter larvae of *B. mori* diapause strain Qiubai. (**E**) The general development process of the silkworm pupae with acetone (left) and KK-42 treated (right). The relative expression level was normalized to the *rp49* gene (**A**–**D**). The lowest expression value was set at 1. The values are the mean ± SEM of three repeat experiments using qRT-PCR. Asterisks indicate significant differences for that group compared with the control group (by Student’s *t*-test, *ns* means no significant difference, * *p* < 0.05; ** *p* < 0.01; *** *p* < 0.001; **** *p* < 0.0001).

To detect the effect of KK-42 on *DH-PBAN* mRNA expression in *A. pernyi* that enters pupal diapause, a monovoltine strain, Yuda no. 1, was used. When 100 μg of KK-42 was applied to diapausing pupae that were woken up by injection of 20E (20 µg), a trigger for diapause termination, the developmental duration of pupae was extended, as previously reported [24]. This indicated that KK-42 used in this study was effective. In the experiment, three time point samples of SG from the control group (24 h, 48 h, and 96 h) and five time point samples of SG from the treated group (24 h, 48 h, 96 h, 144 h, and 192 h) were obtained, due to the rapid development after diapause termination. qRT-PCR detection analysis showed that *DH-PBAN* mRNA expression pattern was similar between the treated and control groups, with a first increasing and then decreasing trend (Figure 2A). The expression of *DH-PBAN* was significantly upregulated in the KK-42-treated group, with a prolonged expression duration, compared to the control group, where *DH-PBAN* mRNA expression remained at a low level. The result indicated that KK-42 could induce up-regulation of the *DH-PBAN* gene in *A. pernyi* entering pupal diapause. To investigate whether this effect is specific to pupal diapause or represents a more general response to KK-42, we next examined its impact in the embryonic diapause model, *B. mori*. This was done to find the answers to the questions through the comparison of different diapause patterns in insects.

### 3.3. KK-42 Upregulates DH-PBAN Gene in the Embryonic Diapause Model B. mori

Building on the findings in *A. pernyi*, we sought to determine if KK-42 exerts a similar effect on *DH-PBAN* in a phylogenetically distant silkmoth with a different diapause strategy. We therefore turned to *B. mori*, which enters diapause during the egg stage. Since the pupal stage of *B. mori* is non-diapausing regardless of embryonic diapause fate, it provides a uniform physiological background to assess the direct effect of KK-42 on *DH-PBAN* expression, independent of the complex hormonal milieu of an ongoing diapause. Here, we used a lower effective dose of KK-42 (2 μg) based on established protocols for *B. mori* [24]. The general development process of the silkworm pupae can be judged by the eye position. The time-course observation showed that eye pigmentation appeared on the third day in the acetone group, while the phenomenon occurred on the sixth day in the KK-42-treated group (Figure 2E). In the experiment, the expression of *DH-PBAN* mRNA continued only in the first two days in strain Nistari that produces non-diapause eggs, but lasted from the first day to the sixth day in strain Qiubai that produces diapause eggs (Figure 2B,C), indicating that the expression duration of *DH-PBAN* mRNA is closely related to the diapause fate of laying eggs. KK-42 induced a significant up-regulation and extended the expression duration of the *DH-PBAN* gene in both Qiubai and Nistari, compared to the control group. The most remarkable difference is the first day after KK-42 treatment, when *DH-PBAN* mRNA expression was similarly low in both KK-42-treated and control groups in Nistari but significantly higher in the KK-42-treated group than in the control group in Qiubai. These results indicated that KK-42 could also induce up-regulation of the *DH-PBAN* gene in *B. mori* with a continuously developing pupal stage.

### 3.4. Effect of KK-42 on DH-PBAN Gene in B. mori Larvae

To investigate the effects of KK-42 on *DH-PBAN* mRNA expression during the larval stage, bivoltine strain Qiubai, a susceptible strain to KK-42, was used. In normal conditions, Qiubai exhibits tetramolter and five larval instars. In the experiment, KK-42 (1 μg) was applied to Qiubai larvae at day 1 of the 4th instar, and the trimolters showing four larval instars were successfully induced with an induction rate of 98%, as previously found [41]. The resulting trimolter silkworms lasted a final fourth instar for 9 days till cocooning, whereas the normal tetramolter silkworms in the control group lasted the fourth instar for 5 days and then entered the fourth molter. The qRT-PCR results showed that *DH-PBAN* mRNA gradually decreased during the fourth instar in the control group (tetramolter silkworm). In the KK-42 treated group (trimolter silkworm), *DH-PBAN* mRNA expression also decreased progressively till the ninth day, showing a highly similar trend with the control group (Figure 2D). However, *DH-PBAN* mRNA expression level was significantly higher in the KK-42-treated group than in the control group. The result indicated that KK-42 could induce up-regulation of the *DH-PBAN* gene in the larval stage of *B. mori*.

### 3.5. RNA-Seq Analysis

In brief, total RNA was extracted from SG tissue of 3 KK-42-treated *B. mori* pupae and 3 *B. mori* pupae controls, and then reverse transcribed into the 6 cDNA libraries with universal primers. 6 cDNA libraries were sequenced and produced raw data (45,839,422 bp and 49,510,692 bp of the mean values of the control and treated groups) and clean reads (45,560,380 bp and 49,176,991 bp of the mean values of the control and treated groups). Clean reads were then mapped onto the silkworm genome database (SilkDB 3.0, https://silkdb.bioinfotoolkits.net/doc/download.html, accessed on 10 January 2026).

**Figure 3:**
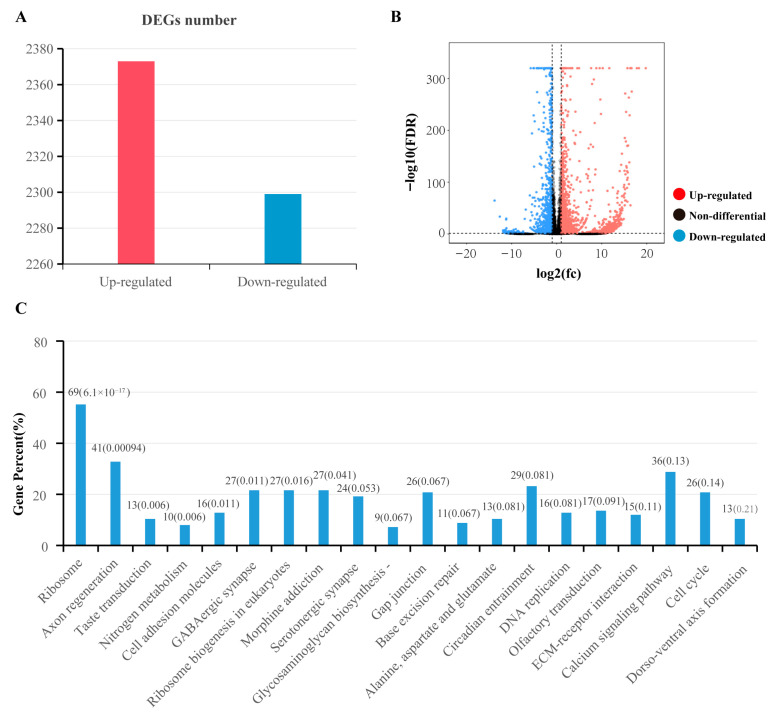
RNA-Seq Analysis of KK-42 treated and normal *B. mori* SG Samples. (**A**) Statistics of DEGs in the *B. mori* SG after KK-42 injection shown in a bar chart. (**B**) Volcano plot illustrating the distribution of DEGs after KK-42 injection. The two vertical dashed lines represent the threshold of |log2| = 1, and the horizontal dashed line represents the threshold of adjusted *p*-value (FDR) ≤ 0.05 for statistical significance. (**C**) KEGG pathway enrichment analysis of pathways in *B. mori* SG after KK-42 injection.

Compared to *B. mori* pupae control, we identified 3572 differentially expressed genes (DEGs), including 2373 up-regulated DEGs and 1199 down-regulated DEGs after KK-42 injection (Figure 3A,B). KEGG pathway enrichment analysis showed that the GABAergic synapse pathway of *B. mori* was significantly regulated after KK-42 was injected (Figure 3C).

### 3.6. Gene Screen and Validation

**Figure 4:**
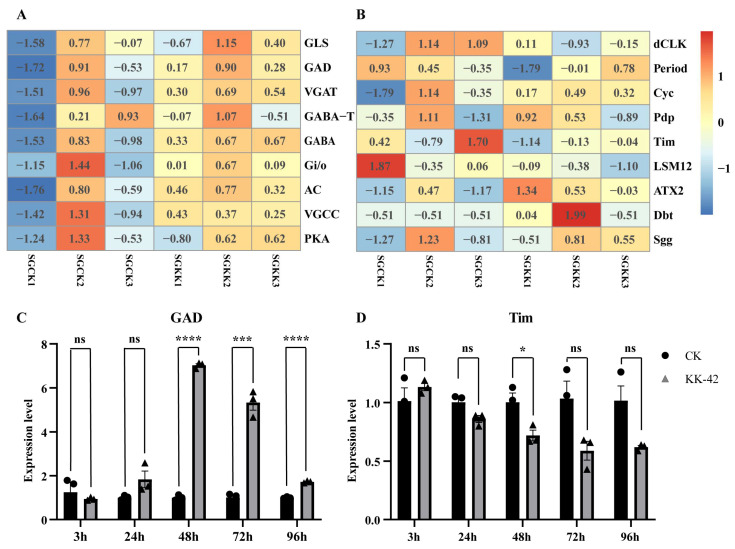
Expression pattern of the genes involved in treatment in the (**A**) GABAergic synapse and (**B**) Circadian signaling pathway in *B. mori*. R software (4.0.3) was used to construct the heatmap; the transcriptome data was dimensionalized using log2. Expression changes of (**C**) *GAD* and (**D**) *Tim* mRNA level in *B. mori* pupae SG after KK-42 (*n* = 3). The relative expression level was normalized to the *rp49* gene. The lowest expression value was set at 1. The values are the mean ± SEM of three repeat experiments using qRT-PCR. Asterisks indicate significant differences for that group compared with the control group (by Student *t*-test, *ns* means no significant difference, * *p* < 0.05; *** *p* < 0.001; **** *p* < 0.0001).

Previous studies have established a direct functional link between GABAergic signaling and the DH-PBAN-mediated diapause regulation pathway in *B. mori* [35], making it the most biologically relevant pathway to our research question. Enrichment analysis revealed the involvement of DEGs in the GABAergic synapse pathway and circadian signaling make a difference (Figure 4A,B), indicating that the genes of this pathway, such as the Bm*GAD* (*glutamic acid decarboxylase*, *GAD*) and *BmTim* (*timeless*, *Tim*), might be affected by the KK-42. Inside, the GABAergic synapse pathway gene *BmGAD* (accession number in SilkDB 3.0: BMSK0000740) was significantly upregulated, and the Circadian pathway gene *BmTim* (timeless, accession number in SilkDB 3.0: BMSK0001959) was suppressed in RNA-seq data.

To verify these, we used q-PCR to detect the expression levels of *BmGAD* and *BmTim* in the KK-42-treated and control silkworm pupal SG samples. The results show that after the KK-42 treated the silkworm after 72 h, the expression of the GABAergic synapse pathway gene *BmGAD* was significantly upregulated, and the circadian gene *BmTim* was significantly suppressed in pupal SG, which were consistent with the result of RNA-seq, and always maintained the expression trend (Figure 4).

## 4. Discussion

In this study, we demonstrate that the imidazole insect growth regulator KK-42 acts as a potent regulator of the *DH-PBAN* neuropeptide gene across two Lepidoptera species with distinct diapause programs. We consistently observed that KK-42 treatment induced significant upregulation and prolonged expression of *DH-PBAN* mRNA in both the pupal diapause model *A. pernyi* and the embryonic diapause model *B. mori*. Beyond this conserved molecular response, a striking phenotypic effect was uncovered in *B. mori*: application of KK-42 to larvae induced a shift from the normal tetramolter (four-instar) to a trimolter (three-instar) developmental pattern. This alteration in molting number represents a fundamental change in developmental timing. Furthermore, transcriptomic analysis pinpointed glutamic acid decarboxylase (*GAD*), a key enzyme in the GABAergic pathway, as being significantly upregulated by KK-42 in *B. mori*.

A recent study has shown that some basal Diptera species [for example, *Aedes aegypti* L. (Diptera: Culicidae) [39] and *Culex quinquefasciatus* Say (Diptera: Culicidae)] possessed the active *DH-PBAN* (also known as hugin) genes, but the higher Diptera species [for example, *D. melanogaster* Meigen (Diptera: Drosophilidae)] have lost two peptides coding sequences for DH and PBAN [37]. Our phylogenetic analysis confirms that while the C-terminal FXPRLamide motif is conserved, the DH-PBAN precursor protein sequences are highly divergent across insect orders, with lineage-specific gene loss events (e.g., loss of DH in *Orthoptera*, loss of both DH and PBAN in higher Diptera) [42]. This highlights the evolutionary plasticity of this neuropeptide system. Within Lepidoptera, however, the conserved response to KK-42 suggests a retained regulatory mechanism. The KK-42-induced shift from tetramolter to trimolter in *B. mori* is a particularly noteworthy finding. The tetramolter/trimolter dimorphism is intrinsically linked to voltinism and diapause induction in silkworms [34]. While we did not establish a direct, one-to-one causal link between this molting shift and altered diapause outcome in the immediate generation, the phenomenon unequivocally shows that KK-42 can reprogram a core aspect of developmental timing. This positions KK-42 as a valuable chemical tool for dissecting the links between larval stage specification, developmental plasticity, and the preparatory physiology for diapause.

The upregulation of *GAD* by KK-42 provides a plausible mechanistic entry point. In the established model for *B. mori* embryonic diapause, GABAergic signaling in the brain-suboesophageal ganglion (Br-SG) complex, mediated by *GAD*, inhibits the release of DH peptide into the hemolymph, thereby promoting diapause initiation [35,43]. Our observation that KK-42 upregulates both *GAD* and *DH-PBAN* mRNA presents an intriguing paradox. We hypothesize that KK-42 may simultaneously stimulate *DH-PBAN* gene transcription while, through elevated GABAergic tone (via increased *GAD*), inhibit the secretion of the translated DH peptide. This could lead to an accumulation of prohormone or hormone within neurosecretory cells, potentially altering the dynamics of its release. This “transcription-secretion uncoupling” hypothesis could explain how KK-42, while elevating *DH-PBAN* mRNA, can ultimately lead to complex or opposite diapause outcomes compared to direct DH application. It must be emphasized that this model remains speculative and requires direct testing through measurement of hemolymph DH titer and functional validation of *GAD*’s role in this context.

Our results provide evidence of the link between KK-42 and the *DH-PBAN* gene. The experiments indicated that KK-42 delayed the pupal development in *A. pernyi* that enters pupal diapause, and induced significant up-regulation of the *DH-PBAN* gene; in *B. mori* pupae, this was also the case. When KK-42 was applied to larvae of *B. mori* Qiubai, we got the trimolters that show significant up-regulation of the *DH-PBAN* gene compared to the normal tetramolters. Some trimolters induced by KK-42 produced diapause eggs, some produced non-diapause eggs, and some produced a mixture of both diapause and non-diapause eggs, confirming that KK-42 can influence the diapause [23]. As for Nistari, a multivoltine non-diapause strain of *B. mori*, we also applied KK-42 to larvae at day 1 of the 4th instar, but we could not get the expected trimolters. In the control group of Nistari pupae, *DH-PBAN* gene expression could be detected in the first two days and then stayed at a very low expression level, which was in line with the previous study [9]; however, KK-42 induced up-regulation of *DH-PBAN* gene, peaked at day 4, and stayed at a low level till day 7. Meanwhile, the expression level of the *GAD* gene increased significantly at 48 h after KK-42 injection, which would promote the GABAergic signal in Br-SG and inhibit DH release into the hemolymph [44]. These results suggested that KK-42 may influence the diapause by upregulating *GAD* gene expression, promoting DH accumulation in SG to prolong the secretion time of the *DH-PBAN* gene in Lepidoptera.

This study has several important limitations that must be acknowledged, as they delineate the boundary between correlation and causation. First, while we measured *DH-PBAN* mRNA levels, we did not quantify the active DH peptide in the hemolymph. The disconnect between transcript abundance and hormone titer is well-documented, and such measurements are essential to validate our hypothesis. Second, the phenotypic assessment of diapause was incomplete: for *A. pernyi*, detailed metrics of pupal diapause duration/termination were not correlated with molecular data; for *B. mori*, the critical outcome of egg diapause incidence in adults derived from KK-42-treated larvae was not systematically quantified. Finally, the functional role of *GAD* and other candidate genes (e.g., *Tim*) identified here remains untested. Consequently, future work must prioritize: (1) radioimmunoassay or ELISA to measure DH hormone titers following KK-42 treatment; (2) comprehensive phenotyping of diapause in both species under the experimental conditions used; and (3) functional genetic approaches (e.g., RNAi, CRISPR-Cas9) in *B. mori* to validate the role of *GAD* in mediating KK-42′s effects on development and diapause. Addressing these points will be crucial to move from the correlative associations reported here to a definitive mechanistic understanding.

## 5. Conclusions

In conclusion, this comparative study demonstrates that the insect growth regulator KK-42 consistently upregulates the expression of the *DH-PBAN* neuropeptide gene in both *Bombyx mori* and *Antheraea pernyi*, and can reprogram larval developmental timing in *B. mori*. These findings establish KK-42 as a valuable molecular tool for manipulating this key neuroendocrine pathway. The associated upregulation of *GAD* suggests a potential mechanistic link to diapause regulation, forming a testable hypothesis for future research. Further investigations focusing on DH hormone titers, detailed diapause phenotyping, and functional validation of candidate genes like *GAD* are needed to fully elucidate the mode of action of KK-42.

## Figures and Tables

**Table 1:** Primers used in this study.

Genes	Sequences (5′-3′)	Length	References
*BmDH*	GCTTTGGCATTGTTCAGTATTT		[34]
	GGCTTCATTGATCGCTTCC		[34]
*ApDH*	GGTAGAAGCATCGGTGACATT		[10]
	CTTTGGGAGTAGCTGGCATATC		[10]
*BmGAD*	CATGATCGGGTGGAAGACTG	72 bp	This study
	AGGAAAGCGTAGAGATTGGAC		This study
*BmTim*	TCAACACCAAATCTCGTAGCG	113 bp	This study
	TGGAGTTTTATGAGACAGCCC		This study
*BmCycle*	AAACGGAAACCATCGTCCTA		[35]
	TTTGTTTCTTGTCGGGAGTG		[35]
*Bmrp49*	GGGTCAATACTTGATGCCCAA		This study
	TCGTCACTCTGATGCTGAGC		This study
*Aprp49*	GGGACAGTATCTGATGCCAAA		This study
	TGGTGACCCTGATGCTTAAC		This study

## Data Availability

Data available on request from the National Genomics Data Center (https://ngdc.cncb.ac.cn, accessed on 19 January 2026). GSA: CRA034903.

## Supplementary Materials

The following supporting information can be downloaded at: https://www.mdpi.com/article/10.3390/biology15070542/s1. Figure S1: Alignment of the protein sequence of the *DH-PBAN* gene of insects. The conserved FXPRLamide neuropeptides are shown. The accession numbers of these *DH-PBAN* sequences can be seen in Figure 1. Two CAPA protein sequences from *Bombyx mori* (NP 001124357) and *Drosophila melanogaster* (FBpp0084880) are used as outgroups.
