# Supplementary material for: Evidence for the Link Between KK-42 and the DH-PBAN Gene in Two Silkmoth Species, with Impacts on Developmental Traits
Source: Biology (Basel). 2026 Mar 28;15(7):542. doi: 10.3390/biology15070542 (PMC13072204; doi:10.3390/biology15070542)
Supplement: Supplementary file 1 [file biology-15-00542-s001.zip › biology-4155723-supplementary.pdf]

```
Antheraea pernyi              ------------------------------------MYFKNQNMFKLFII
Samia ricini                  ------------------------------------MSPKSQNMCRLFLI
Manduca sexta                 ---------------------------------------MSQILIEFIAL
Heliothis peltigera           ------------------------------------MYVQRKCLFLLAVF
Heliothiv virescens           ------------------------------------MFYQTQLFVFLAVF
Bombyx mori                   -------------------------------------MYKTNIVFNVLAL
Bombyx mandarina              -------------------------------------MYKTNIVFNVLAL
Helicoverpa armigera          -------------------------------------MFQTQLFVFLAVF
Helicoverpa zea               ------------------------------------MFNQTQLFVFLAVF
Spodoptera exigua             -------------------------------------MFSSTIFFAISVT
Ostrinia nubilalis            --------------------------------------MIFNFKLLFFVL
Maruca vitrata                -----------------------------------MSSLNFKVLFYIFFL
Omphisa fuscidentalis         -------------------------------------MSIFNLKFVLSIF
Pieris brassicae              ------------------------------------MALDRIMFTVLLFV
Danaus plexippus              ------------------------------------MGCIWYFLLSFTIL
Aedes aegypti                 ---------------------------------------MFRLYFFFNVI
Culex quinquefasciatus        ---------------------------------------MFKLYFVFNLV
Anopheles gambiae             ---------------------------------------MSRFYFFFNLI
Solenopsis geminata           ------------------------------------MIVTRNSVNRATIV
Apis mellifera                ----------------------------MIGFAVFSSFNRFTTIFVCVLL
Tribolium castaneum           --------------------------------------MERFILINWTVL
Aethina tumida                ------------------------------------MKQSTKINFALIFM
Locusta migratoria            --------------------------------------MAPAVAAALLLL
Schistocerca gregaria         --------------------------------------MSAPVAAALIVL
Cryptotermes secundus         --------------------------------MRTGFSTEQHLVQSIVLL
Zootermopsis nevadensis       --------------------------------MRTDFSTQQHLIHTIVLL
Brachyptera putata            ------------------------------------MMCSVHCTMLVLWG
Bemisia tabaci                --------------------------------MDKVFCFRNFEMGYLIVA
Stenopsyche tienmushanensis   MSAEREAFLCRIRGPISGPLGHRDLVASSGAFPAFKAMKMNRSIIFAVIL
Bombyx mori                   ------------------------------------MQSTMKLLTIITLI
Drosophila melanogaster       -----------------------------------MKSMLVHIVLVIFII

                                                      ε-SGNP
Antheraea pernyi              SV--FGLVLA----------------------------------------
Samia ricini                  SL--FSLVLA----------------------------------------
Manduca sexta                 CL--ICVVTG----------------------------------------
Heliothis peltigera           TI--SSVSGN----------------------------------------
Heliothiv virescens           AT--TSVLGN----------------------------------------
Bombyx mori                   AL--FSIFFAS---------------------------------------
Bombyx mandarina              AL--FSIFFAS---------------------------------------
Helicoverpa armigera          TT--SSVLGN----------------------------------------
Helicoverpa zea               TT--SSVLGN----------------------------------------
Spodoptera exigua             CV-----LAN----------------------------------------
Ostrinia nubilalis            SL--FCALAT----------------------------------------
Maruca vitrata                CG--VSV-------------------------------------------
Omphisa fuscidentalis         AL--FCGFATA---------------------------------------
Pieris brassicae              TY--SLVLVC----------------------------------------
Danaus plexippus              LF--TLHLIG----------------------------------------
Aedes aegypti                 CI--FLAIRS--------------------AIGGEVPDATEQK-------
Culex quinquefasciatus        CI--YLAIKSAFSAEVPDPNEQKI--------------------------
Anopheles gambiae             CL--YLAIKSALSAELDTNDQKYADLRTTGRGESPDSTGPD---------
Solenopsis geminata           CI--MAMLLC---------LGSRASGEYESREIGSNGGSSESR-------
Apis mellifera                CVVYLLSYAS----------------------GEYDGRDSSSG-------
Tribolium castaneum           CV--AVLFFET---------------------------------------
Aethina tumida                SL--IILEIC----------------------------------------
Locusta migratoria            CS--ASVAAAHGGGGS---WVSRREGDFTPRLGR-ESAEQGGGVSAWQGG
Schistocerca gregaria         CS--ATVAAGHGGGGTGSGWVGRREGDFTPRLGR-ESAEQGAA-PQWQSA
Cryptotermes secundus         LL--VTFLIS------------------CNVSGLRLGTDPIGD-------
Zootermopsis nevadensis       CL--VVALAS------------------CD--GFRLSSDPLED-------
Brachyptera putata            AV--FATSKG----------------------------------------
Bemisia tabaci                VI--TSALIS---------FCQ--GIQQMDPRAISRSDILEEA-------
Stenopsyche tienmushanensis   CY--FLDSCG------------------------KIYGEPIS--------
Bombyx mori                   SITFNSSHQS----------------------------------------
Drosophila melanogaster       AE--FSTAET----------------------------------------

                                         ζ-SGNP                    η-SGNP
Antheraea pernyi              -------------------SNDIKDEGD-KGAHSDRGS------------
Samia ricini                  -------------------TNDVKDEGD-RGAHSDRGS------------
Manduca sexta                 -------------------SNDIKDEGD-RGAHSDRGA------------
Heliothis peltigera           -------------------NNDVKDGAD-RGAHSDRGG------------
Heliothiv virescens           -------------------NNDDKDGAA-SGAHSDRLG------------
Bombyx mori                   -------------------CTDMKDESD-RGAHSERGA------------
Bombyx mandarina              -------------------CTDMKDESD-RGAHSERGA------------
Helicoverpa armigera          -------------------NNDVKDGGAASGAHSDRLG------------
Helicoverpa zea               -------------------NNDVKDGAA-SGAHSDRLG------------
Spodoptera exigua             -------------------NNEIKDGGSDRGAHSDRAG------------
Ostrinia nubilalis            -------------------AVDDLKDDVDRGA-SDRGT------------
Maruca vitrata                -------------------ALDDSKDEVDRGA-SDRGG------------
Omphisa fuscidentalis         -------------------VDDLKDEADRGA--SDRGT------------
Pieris brassicae              -------------------GVDSKDDIQDRGAHSDRGG------------
Danaus plexippus              -------------------ASDMKDDNLDRGAHSDRGG------------
Aedes aegypti                 -------------------INNFLASGKDSEDLSKRAAA-----------
Culex quinquefasciatus        -------------------SNYLSSAGGSDDDVGKRASA-----------
Anopheles gambiae             -------------------SDTLRRDDGAEGLNKRAAA------------
Solenopsis geminata           -------------------SPSNDFGSCIDGKCIKRTSQDIASG------
Apis mellifera                -------------------SNNDRAPSNEFGSCTDGKCIKRTSQDITSG-
Tribolium castaneum           -------------------VLSTPHESSVPNERNDDSKETY---------
```

```
Aethina tumida                 -------------------CAESHHGNLSADKPEHEKHASE---------
Locusta migratoria             EPQQEEQVLAGPFVPRLGR----------------GAVPAAQFSPRLGRR
Schistocerca gregaria          ----EEQVLSGPFVPRLGR----------------GAAPAAQFSPRLGRR
Cryptotermes secundus          -------------------AILLGLDGARDDGPMVKKSDPQ----VYG--
Zootermopsis nevadensis        -------------------GLLLGLEGLGDDPLAAKRGEPE----VTG--
Brachyptera putata             -------------------SPLLRYPAASDVDVSDEEGSTMVKRGPVNPG
Bemisia tabaci                 -------------------MLLGLEVGASDGVDSGRKRSGK---ASFNRA
Stenopsyche tienmushanensis    -------------------VEDMKMGANIEDKFDQDFEKRGSPGG-----
Bombyx mori                    -------------------GAKLRPDGVLN--------------------
Drosophila melanogaster        -------------------DHDKNRRGANMG-------------------

                                  DH
Antheraea pernyi               -LWFGPRLGKRSLSLSSEDDRQKFFRLLEAADTLKFYYDQLPYYQKQADE
Samia ricini                   -LWFGPRLGKRSLSLTGEDDRQKFLRLLEAADALKFYYDQLPYYQRQPDE
Manduca sexta                  -LWFGPRLGKRSLKLSSEDDRQAFFRLLESADTLKYYYDQLPYYERQIDE
Heliothis peltigera            -LWFGPRLGKRSLRIATEDNRQAFFKLLEAADALKYYYDQLP-YEMQADE
Heliothiv virescens            -LWFGPRLGKRSLRISTGDNRQAFFKLLEAADALKYYYDQLP-YEMQADD
Bombyx mori                    -LWFGPRLGKRSMKPSTEDNRQTFLRLLEAADALKFYYDQLP-YERQADE
Bombyx mandarina               -LWFGPRLGKRSMKPSTEDNRQTFLRLLEAADALKFYYDQLP-YERQADE
Helicoverpa armigera           -LWFGPRLGKRSLRISTEDNRQAFFKLLEAADALKYYYDQLP-YEMQADE
Helicoverpa zea                -LWFGPRLGKRSLRISTEDNRQAFFKLLEAADALKYYYDQLP-YEMQADE
Spodoptera exigua              -LWFGPRLGKRSLRISTEDNRQAFFKLLEAADALKYYYDRLP-YEMQADE
Ostrinia nubilalis             -LWFGPRLGKRSLRISNDDNKQTFLRLLEAADALKFYYDQLPFYESQADD
Maruca vitrata                 -LWFGPRLGKRSLRVNNDDNRQTFLRLLEAADALKYYYDQLPFYDSQVDD
Omphisa fuscidentalis          -LWFGPRLGKRSLRISNDDNRQTFLRLLEAADALKYYYDQLPFYESRADD
Pieris brassicae               -VWFGPRLGKRSLRL-GDDSKATLLRLIESADNLRYYYDQLP-FELQSDV
Danaus plexippus               -VWFGPRLGKRSLQLLDDSDSQTFVRLLEAAEALKYYYDQMS-YQMQADA
Aedes aegypti                  -MWFGPRLGKRTIAS------ELHDEMMDEIDDNPLYY--------SGES
Culex quinquefasciatus         -MWFGPRLGKRTVTP------ELHDDLLEELDGSPMFY--------QGEA
Anopheles gambiae              -MWFGPRLGKRTIAA------DLHDDLVEEFDAEPLGY--------AGEP
Solenopsis geminata            -MWFGPRLGKRYKSDEKQELSSEIEILANALDGVR---------------
Apis mellifera                 -MWFGPRLGRRRRA----DRKPEINSDIEA-------------FANAFEE
Tribolium castaneum            -FWFGPRLGRKKRNSSNDDLYQDMQKEELVSL-----------TDALQDV
Aethina tumida                 -LWHGPKLGRKKRNPSDDLFREELEQKEQANL-----------FDMLQDT
Locusta migratoria             -----------DPPVDGPLVW-LPLQVSPRLARRRQQP------------
Schistocerca gregaria          -----------DPPADG-LVW-LPL--VPRLGRRRPLP---------APA
Cryptotermes secundus          -MWFGPRLGRRERRSVDEILDDAGDGRVEEV------------LQLLKET
Zootermopsis nevadensis        -MWFGPRLGRREKRSVDDFPEDVADIRVEEV------------MELLKDT
Brachyptera putata             -MWFGPRLGRRERRGADDLQEEGDGGKVDSS------------LVELLDS
Bemisia tabaci                 DLWFGPRLGRKKRNSEEELGPEIPDKDEESILEF-----------IKSSS
Stenopsyche tienmushanensis    -MWFGPRLGKRSKEYVNDHNSASLQRILEAM-----------------SS
Bombyx mori                    -LYPFPRVGRASYRTWQIPINDVYL------------------------D
Drosophila melanogaster        -LYAFPRVGRSDPSLANSLRDGLEAGVLDGI-----------YGDASQED
                                  .
                                          α-SGNP                        β-SGNP
Antheraea pernyi               P-ETKVTKKVIFTPKLGRSIGDIYQEKRT-YENV----EFTPRLGRRLSD
Samia ricini                   P-ETKVTKKVIFTPKLGRRASNAYQEKRT-YENV----EFTPRLGRRLTE
Manduca sexta                  P--AKVTKKVIFTPELGRSLDDSTQEKRVFYENF----EFTPRLGRRISE
Heliothis peltigera            P-ETRVTKKVIFTPKLGRSIA---YEDKS-FENV----EFTPRLGRRLAD
Heliothiv virescens            P-ETRVTKKVIFTPKLGRSLS---YDDKS-FENV----EFTPRLGRRLAD
Bombyx mori                    P-ETKVTKKIIFTPKLGRSVANP----RT-HESL----EFIPRLGRRLSE
Bombyx mandarina               P-ETKVTKKIIFTPKLGRSVAKP----QT-HESL----EFIPRLGRRLSE
Helicoverpa armigera           P-ETRVTKKVIFTPKLGRSLA---YDDKS-FENV----EFTPRLGRRLSD
Helicoverpa zea                P-ETRVTKKVIFTPKLGRSLA---YDDKS-FENV----EFTPRLGRRLSD
Spodoptera exigua              P-ETRVTKKVIFTPKLGRSLA---YDDKV-FENV----EFTPRLGRRLSD
Ostrinia nubilalis             P-ETRVTKKVVFTPKLGRSID-VYPEKRT-FENV----EFTPRLGRRLPE
Maruca vitrata                 P-ETRVTKKVVFTPKLGRSIGGVFQDKK--YDNV----EFTPRLGRRIPD
Omphisa fuscidentalis          P-ETRVTKKVIFTPKLGRSMD-GYSDKRT-YENV----EFTPRLGRRLPE
Pieris brassicae               G-Q----DKIIFTPKLGREID-----ERL-LNDV----EFTPRLGRRKIN
Danaus plexippus               P-Q-KVIKKVIFTPKLGRALD--QYSERM-AGNI----DFTPRLGRKLPE
Aedes aegypti                  P-QRVASEIAQGTPYVVLLLT--GRVLR--QPQPVFYHSTTPRLGRRDAS
Culex quinquefasciatus         P-QRLAADIAQGSPYLVLLVTAAGGRIA--KPQPVFYHSATPRLGRR---
Anopheles gambiae              P-QKLATELVQGAPYMVLLVTAKPRKPQPIFYH-----TTSPRLGRRDSV
Solenopsis geminata            --WAVITIPASDK------------------RQP----QFTPRLGRGSGE
Apis mellifera                 PHWAIVTIPETEK-----------------RQIT----QFTPRLGRESGE
Tribolium castaneum            P-WAIIAVNDLLEGK---------------RHVV----NFTPRLGRESGE
Aethina tumida                 P-WTVVAVGDGKR------------------HVS----SFTPRLGRELQD
Locusta migratoria             ---------------------------------------FVPRLGRDSGD
Schistocerca gregaria          P-APAAP--------------------------------FVPRLGRDSSE
Cryptotermes secundus          P-WVLVPLKGNKR------------------QTG----SFIPRLGRDSKE
Zootermopsis nevadensis        P-WALLPLRGGKR------------------HIE----GFVPRLGRDSNE
Brachyptera putata             P-WALIALKGGRR------------------HTM----SFTPRLGRDSGE
Bemisia tabaci                 P-WVLIPLKEKANT-----------------RSM----NYTPRLGRSSKE
Stenopsyche tienmushanensis    P-EYSASNYGMSG------------------SKR----RFTPRLGRDSSE
Bombyx mori                    -YEPVEKRQL----------------------------YAFPRVGRGGPP
Drosophila melanogaster        -YNEADFQKKAS-------------------GL-----VAFPRVGRGDAE

                                                               PBAN
Antheraea pernyi               --DMPATPKDQE--MY--HQDPEQVDT-RTRYFSPRLGRTI---------
Samia ricini                   --DMPATPTDQE--MF--DQDPEQIDT-RTRYFSPRLGRTM---------
Manduca sexta                  --DMPATPSDQEYPMY--HPDPEQIDT-RTRYFSPRLGRT----------
Heliothis peltigera            --DMPATPADQE--IY--RQDPEQIDS-RTKYFSPRLGRTM---------
Heliothiv virescens            --DMPATPADQE--MY--RQDPEQIDSRRTKYFSPRLGRTM---------
Bombyx mori                    --DMPATPADQE--MY--QPDPEEMES-RTRYFSPRLGRTM---------
Bombyx mandarina               --DMPATPADQE--IY--QPDPEVMES-RTRYFSPRLGRTM---------
Helicoverpa armigera           --DMPATPADQE--MY--RQDPEQIDS-RTKYFSPRLGRTM---------
Helicoverpa zea                --DMPATPADQE--MY--RQDPEQIDS-RTKYFSPRLGRTM---------
```

```
Spodoptera exigua               --DMPATPADQE--LY--RPDPDQIDS-RTKYFSPRLGRTM---------
Ostrinia nubilalis              --KVPVTPSDSHDEVYSFKPDMEEIIS-RHNYFSPRLGRTL---------
Maruca vitrata                  --ALPVTPSDDD--VYSFKPDSGEVDR-RTSYFNPRLGRKV---------
Omphisa fuscidentalis           --KLSVTPSDSHDAVYSFKPEMSELDS-RNNYFSPRLGRTV---------
Pieris brassicae                --QALPTVTDEE--SY--RQDQMLMNN-RPNQFSPRLGRNY---------
Danaus plexippus                --RTPTTSSDED---S--IQDAIAANR-RPSYFSPRLGRNY---------
Aedes aegypti                   ----------------------SSNENNSRPPFAPRLGRNL---------
Culex quinquefasciatus          --------------------DASAQDVHSRPPFAPRLGRNL---------
Anopheles gambiae               ------------------------GENHQRPPFAPRLGRNL---------
Solenopsis geminata             --DLFS------------YGDAYEVDEDDHPLFVPRLGRRL---------
Apis mellifera                  --DYFSYGFPKD------QEELYTEEQIYLPLFASRLGRRV---------
Tribolium castaneum             --EFVNNAPEDRWLQN--HETSGEMLYQRSPPFAPRLGRHSS--------
Aethina tumida                  --D---------------FGSNMEFELSGRSPFSPRLGKRMT--------
Locusta migratoria              --------------------------EWPQQPFVPRLGRRLHQNGM----
Schistocerca gregaria           ---------------------------DWAQPFVPRLGRRLQQYGM----
Cryptotermes secundus           --E---------------EEDPDAMEQ-RSPPFAPRLGRRLV--------
Zootermopsis nevadensis         --D----------------EDADMMEQ-RSPPFAPRLGRRLV--------
Brachyptera putata              --EEYS------------DSGLRLVDM-RSPPFSPRLGRRLV--------
Bemisia tabaci                  -----------------EEEDFIPEIT-RSTPFVPRLGKRR--NNQ----
Stenopsyche tienmushanensis     ---------------------QEIIGPSKSVDFIPRLGRELTPGLLRAIL
Bombyx mori                     -SDRNEPHDDLLGLH---LDDP-------GMWFGPRLGRSLKNGDD----
Drosophila melanogaster         LRKWAHLLALQQV-----LDKRTGPSASSGLWFGPRLGKRSVDAK-----

                                                             γ-SGNP         δ-SGNP
Antheraea pernyi                -----------------------------TFSPRLGREL--SYD------
Samia ricini                    -----------------------------TFSPRLGREL--NYD------
Manduca sexta                   -----------------------------HFSPRLGREL--SYD------
Heliothis peltigera             -----------------------------NFSPRLGREL--SYD------
Heliothiv virescens             -----------------------------NFSPRLGREL--TYD------
Bombyx mori                     -----------------------------SFSPRLGREL--SYD------
Bombyx mandarina                -----------------------------SFSPRLGREL--SYD------
Helicoverpa armigera            -----------------------------NFSPRLGREL--SYD------
Helicoverpa zea                 -----------------------------NFSPRLGREL--SYD------
Spodoptera exigua               -----------------------------NFSPRLGREL--SYE------
Ostrinia nubilalis              -----------------------------NFSPRLGRELDSSYD------
Maruca vitrata                  -----------------------------SFSPRLGREL--TYD------
Omphisa fuscidentalis           -----------------------------NFSPRLGREL--SYD------
Pieris brassicae                -----------------------------NFSPRLGREL--TYD------
Danaus plexippus                -----------------------------NFSPRLGREL---YE------
Aedes aegypti                   -----------------------------PFSPRLGRSF--GAP------
Culex quinquefasciatus          -----------------------------PFSPRLGRSF-----------
Anopheles gambiae               -----------------------------PFSPRLGRSYNGGYP------
Solenopsis geminata             ---------------------------PWIPSPRLGRQL-----------
Apis mellifera                  ---------------------------PWTPSPRLGRQL---HN------
Tribolium castaneum             -----------------------------PFSPRLGREN--DRN------
Aethina tumida                  -----------------------------PFSPRLGR-------------
Locusta migratoria              -----------------------------PFSPRLGRDA-----------
Schistocerca gregaria           -----------------------------PFSPRLGRDA-----------
Cryptotermes secundus           -----------------------------PFRPRMGRDR-LPYDVYSPRL
Zootermopsis nevadensis         -----------------------------PFRPRMGRDR-LPHDVYSPRL
Brachyptera putata              -----------------------------PFSPRLGRERDV---VYAPRL
Bemisia tabaci                  -----------------------------IFSPRLGRSDLYYSP------
Stenopsyche tienmushanensis     LNANDLNTVLYGRAIGEDRGPTLTESKVPPFAPRLGREMYLPLQ------
Bombyx mori                     --------------------------DVVNQNEDGRSEREQID-------
Drosophila melanogaster         --------------------------SFADISKGQKEL----N-------

Antheraea pernyi                --------------YP-INVRVARSTNATAN---------------
Samia ricini                    --------------YP-INVRMARSAHNTTDIN-------------
Manduca sexta                   --------------MP-TNVRVARSTNKTLN---------------
Heliothis peltigera             -------------MLP-NKIRVARSTNKTRST--------------
Heliothiv virescens             -------------MLP-NKIRVVRSTNKTRST--------------
Bombyx mori                     --------------YP-TKYRVARSVNKTMDN--------------
Bombyx mandarina                --------------YP-TKYRVARSVNKTMDN--------------
Helicoverpa armigera            -------------MIP-NKIRVVRSANKTRST--------------
Helicoverpa zea                 -------------MMP-NKIRVVRSTNKTRST--------------
Spodoptera exigua               -------------MLP-SKLRMVRSTNRTQST--------------
Ostrinia nubilalis              -------------IYP-EKIRLARSANLTKT---------------
Maruca vitrata                  -------------IYP-EKIRLARSANDSKAT--------------
Omphisa fuscidentalis           -------------IYP-EKIRLARSINLTKT---------------
Pieris brassicae                -------------LYP--SVRVSRSVNNSKTN--------------
Danaus plexippus                -------------LYPEERVRVARSVNGTNSK--------------
Aedes aegypti                   --------------VV-DNFAY------------------------
Culex quinquefasciatus          --------------------GGAQTVDSFGF---------------
Anopheles gambiae               --------------LP-FQFAY------------------------
Solenopsis geminata             -----------------------RNVLRKL----------------
Apis mellifera                  -------------IVDKPRQNFNDPRF-------------------
Tribolium castaneum             -------------LFS------------------------------
Aethina tumida                  ----------------------------------------------
Locusta migratoria              -----------------AEQQPADE---------------------
Schistocerca gregaria           ---------------P-GQLQADEQ---------------------
Cryptotermes secundus           GR-----------SVQPSQPQGKKEAPQH-----------------
Zootermopsis nevadensis         GR-----------SVPHEKKQTPPHH--------------------
Brachyptera putata              GR-----------QAATTLDNNTH----------------------
Bemisia tabaci                  -------------RSLRSAPPKQQQ---------------------
Stenopsyche tienmushanensis     ---SDFYGLPRGLTIKDGNSRIARSADNTKIGHNKCAEVLMKSSKP
Bombyx mori                     ------------QIA-HEERMKRRSKLL------------------
[illegible]
```

Figure S1. Alignment of the protein sequence of the DH-PBAN gene of insects. The conserved FXPRLamide neuropeptides are shown. The accession numbers of these DH-PBAN sequences can be seen in Figure 1. Two CAPA protein sequences from *Bombyx mori* (NP 001124357) and *Drosophila melanogaster* (FBpp0084880) are used as outgroups.
